# Identification of Reference Genes for Quantitative Gene Expression Studies in Three Tissues of Japanese Quail

**DOI:** 10.3390/genes10030197

**Published:** 2019-03-04

**Authors:** Anaïs Vitorino Carvalho, Nathalie Couroussé, Sabine Crochet, Vincent Coustham

**Affiliations:** BOA, INRA, Université de Tours, 37380 Nouzilly, France; anais.carvalho@inra.fr (A.V.C.); nathalie.courousse@inra.fr (N.C.); sabine.crochet@inra.fr (S.C.)

**Keywords:** Japanese quail, reference gene, RT-qPCR, gene expression

## Abstract

RT-qPCR is the gold standard for candidate gene expression analysis. However, the interpretation of RT-qPCR results depends on the proper use of internal controls, i.e., reference genes. Japanese quail is an agronomic species also used as a laboratory model, but little is known about RT-qPCR reference genes for this species. Thus, we investigated 10 putative reference genes (*ACTB*, *GAPDH*, *PGK1*, *RPS7*, *RPS8*, *RPL19*, *RPL32*, *SDHA*, *TBP* and *YWHAZ*) in three different female and male quail tissues (liver, brain and pectoral muscle). Gene expression stability was evaluated with three different algorithms: geNorm, NormFinder and BestKeeper. For each tissue, a suitable set of reference genes was defined and validated by a differential analysis of gene expression between females and males (*CCNH* in brain and *RPL19* in pectoral muscle). Collectively, our study led to the identification of suitable reference genes in liver, brain and pectoral muscle for Japanese quail, along with recommendations for the identification of reference gene sets for this species.

## 1. Introduction

Japanese quail (*Coturnix japonica*) is the smallest avian species farmed for egg and meat production and is a popular source of proteins in the world [1,2]. In addition to being an established model for embryology studies [3], Japanese quail is also a popular laboratory model [4] especially for behavior [5], genetics and genomics studies [6,7]. Females are sexually mature at 5–6 weeks of age, allowing the production of 3 to 4 generations per year [2,8]. The complete Japanese quail genome sequence was recently released (2016, The International Quail Genome Consortium and McDonnell Genome Institute, Washington University School of Medicine) and inbred laboratory lines are available, thus facilitating genome-wide studies. Together, Japanese quail can be considered as an economic and multipurpose animal model for research [1,9].

Since its description in 1992 [10], real-time PCR (qPCR) has been the most common technique to quantify nucleic acid abundance for molecular diagnostics and life science research [11,12,13,14]. Indeed, given its accuracy, reproducibility, low cost and speed as well as reduced labor, qPCR can permit the detection and quantification of very limited copies of nucleic acid [10,11,12,13,14]. Thus, associated with reverse-transcription (RT), this technique became the gold standard to evaluate gene expression. Furthermore, now that transcriptome studies are less expensive and thus more accessible [15], RT-qPCR is routinely used as validation tool to confirm gene expression analysis observed in microarray and RNA-seq experiments [16]. One limitation, however, is the necessity of using reliable reference genes that are required for the interpretation of RT-qPCR results [17]. 

RT-qPCR quantification depends on various parameters in the workflow, especially RNA quality and qPCR efficiency. To estimate these parameters and avoid bias, the use of an internal reference is necessary to compare several samples and various experimental conditions (time points, tissues, treatments, etc.). Thus, a reference gene is defined as non-variant gene between all samples and all experimental conditions [17]. Among published studies, the *GAPDH* gene is frequently used as a reference gene [12]. Nowadays, the normalization to a single reference gene is generally admitted as suboptimal for accurate interpretation and the combination of multiple reference genes to mimic the ideal reference and limit the natural variation is preferred [18,19,20,21,22]. 

The identification of multiple reference genes has been performed in various animal species including pig [23], cattle [24,25,26], dog [27] and avian species (in particular, chicken) [28,29,30,31]. Concerning quails, although a RT-qPCR normalization based on several genes was already published, this study was limited to an embryonic tissue, the blastoderm [32]. Thus, there is a lack of information regarding suitable reference genes in adult quails. In the present study, we investigated 10 potential reference genes in 3 tissues (liver, pectoral muscle and brain) in both sexes. To estimate the suitability as reference gene of each candidate, we used the statistical tools geNorm [22], NormFinder [19] and BestKeeper [18]. Finally, we used reference genes to normalize the expression of differentially expressed genes between both sexes. Collectively, our data allowed the identification of a set of reference genes suitable for the analysis of gene expression in the liver, brain and pectoral muscle of Japanese quail. 

## 2. Materials and Methods 

### 2.1. Animal Sample Collection

All experiments were carried out in accordance with the legislation governing the ethical treatment of birds and were approved by the French Ministry of Higher Education and the Val-de-Loire Animal Ethics Committee (authorisation N° APAFIS#4606-2016032111363124). They were performed in the INRA UE1295 PEAT experimental facilities (Poultry Experimental Unit of Tours, Agreement N° C37-175-1).

Cons DD quails (INRA) were raised in standard conditions. At 35 days of age, 10 females and 10 males were sacrificed and liver, pectoral muscle (*Pectoralis major*) and brain (excluding hypothalamus) were immediately sampled and frozen in liquid nitrogen. Samples were stored at −80 °C before the analyses. 

### 2.2. RNA Extraction

All tissue samples were ground in liquid nitrogen and 25 mg of powder was used for RNA extraction using a NucleoSpin^®^ RNA kit (Macherey-Nagel, Bethlhem, PA, USA), according to the manufacturer’s instructions for the isolation of RNA from hard-to-lyse tissues. RNA integrity and concentration were measured by migration on 1% agarose gel and by quantification on a Nanodrop ND-1000 spectrophotometer, respectively. The RNA purity was verified by the analysis of the A260/A280 ratios, which were all between 2.10 and 2.18. To avoid DNA contamination as recommended by the Minimum Information for Publication of Quantitative Real-Time PCR Experiments (MIQE) guidelines [17], DNAse (Ambion by Life Technologies, Carlsbad, CA, USA) treatment was performed on 0.545 µg of total RNA according to the manufacturer’s instructions and then RNA integrity was checked by migration on 1% agarose gel.

### 2.3. Reverse Transcription and Quantitative Real-Time PCR

RT-qPCR experiments were performed following the MIQE guidelines [17].

cDNA was synthesized from 0.5 µg of total RNA using the Superscript II enzyme (Invitrogen, California, CA, USA) and random hexamer primers (Promega, Fitchburg, WI, USA) in a final volume of 20 μL, following the manufacturer’s instructions. 

Gene expression was evaluated in each tissue independently. Primer sequences were designed following the MIQE guidelines [17] with NCBI Primer blast (https://www.ncbi.nlm.nih.gov/tools/primer-blast/index.cgi?LINK_LOC=BlastHome; Table 1). All primers were designed and tested for an annealing temperature of 60 °C. The absence of primer secondary structure was checked using OligoEvaluator (Sigma-Aldrich, Saint-Quentin Fallavier, France, http://www.oligoevaluator.com/). qPCR reactions were performed in a final volume of 10 µL (2.5 µL of RT dilution, 2 µL of water, 5 µL of Takyon mix, 0.25 µL of each primer at 10 µM) with a Takyon qPCR kit (Eurogentec, Liège, Belgium) and using a LightCycler^®^ 480 Instrument II system (Roche, Basel, Switzerland) in white 384-well plates (4titude, Surrey, UK) sealed by adhesive films. A standard curve analysis was performed by pooling all cDNA samples (serial dilution from 1/10 to 1/5000 in water; eight measured points) to calculate the PCR efficiency and the correlation coefficient of each primer pair in each tissue independently. Reactions were performed on two technical replicates. A denaturation step (5 min at 95 °C) was followed by an amplification step with 45 cycles of 10 s at 95 °C, 20 s at 60 °C and 10 s at 72 °C. To ensure the presence of only one amplicon, melting curves were produced by gradually increasing the reaction temperature from 65 °C to 95 °C (5 s at 95 °C, 1 min at 65 °C, heating to 95 °C at a rate of 0.11 °C/s) (Supplementary Figures S1–S3). Furthermore, the presence of a unique amplicon and its size were checked on a 2% agarose gel. The amplicon sequence was verified by Sanger sequencing (Genewiz, Leipzig, Germany) and then blasted on the NCBI database of quail transcripts (genome: Coturnix japonica 2.0). To ensure the absence of primer dimers, negative control was analyzed as a no-template sample for which cDNA was replaced by water. 

The analysis of standard curves revealed that a 200-fold dilution of RT was suitable for the gene expression analysis of all primer pairs. qPCR reactions for gene expression evaluation were performed in the same conditions as those previously described (including the presence of a no-template sample). Reactions were performed on three technical replicates. The Cq (quantification cycle) was determined as the average of the three technical replicates [17]. As recommended [17], no Cq >40 was included in the analysis. Gene expression evaluation was considered as correct when the Cq identified for each sample was included in the linear dynamic range determined for each primer pairs, in each considered tissue. A pooled sample (i.e., a pool of all samples in a given tissue) at the same dilution of cDNA samples (i.e., 200-fold diluted) was used to calculate gene expression with the comparative Cq (ΔCq) method: 2^(Cq pool-Cq sample)^.

### 2.4. Gene Stability Analyses

A gene was considered stable if its expression was equivalent in all the studied samples [17,18,19,21,22]. The gene stability was investigated with three different algorithms: geNorm [22], NormFinder [19] and BestKeeper [18]. The analyses with geNorm (version 3.5) and NormFinder (version 0.953) were performed on ΔCq and the analysis with BestKeeper (version 1.0) was performed on raw Cq as input. As recommended, a gene was considered stable when its stability value was <1.5 with geNorm [22] and <0.25 with NormFinder [19]. Each tissue was studied independently. The normalization factor was automatically calculated by geNorm based on the best combination of reference genes or manually calculated as the geometric mean of the combination of the two most stable genes with NormFinder. 

### 2.5. Statistical Analyses

Statistical analyses were performed using RStudio [33] (R version 3.5.1). The impact of sex on gene expression was investigated using a *t*-test. A difference of gene expression between sexes was considered significant for *p*-values ≤0.05.

## 3. Results

### 3.1. Primer Design, Real-Time qPCR Experiment and PCR Efficiency

To identify RT-qPCR reference genes, we designed primers for *ACTB*, *GAPDH*, *PGK1*, *RPL19*, *RPL32*, *RPS7*, *RPS8*, *SDHA*, *TBP* and *YWHAZ* based on annotated quail transcripts (NCBI database Coturnix japonica 2.0, Table 1). Determination of the linear dynamic range of Cq was performed for each primer pair using serial dilutions from 1/10 to 1/5000 on a pool of cDNA for each tissue (Table 2). All genes were expressed in the three tissues and detected between 14 and 34 Cq (Table 2). qPCR efficiencies did not differ substantially between genes and were close to 100% with acceptable R² values varying from 0.95 to 0.99 (Table 2). qPCR specificity was verified by melting curve analysis (Supplementary Figures S1–S3), agarose gel migration and Sanger sequencing followed by BLAST. No primer dimers was observed in the no-template sample. Finally, the expression of all these genes in the samples was comprised in the linear dynamic range (Table 2). 

### 3.2. Impact of Sex on the Expression of Putative Reference Genes

The impact of the sex on reference genes was analyzed in each tissue separately. The expression of each putative reference gene was calculated with the ΔCq method using a pool of all cDNA samples relative to one specific tissue as a reference. In liver and brain, no impact of the sex was observed for all genes tested (Table 3). *RPL19*, *RPL32* and *RPS8* were significantly impacted by the sex in the pectoral muscle (with respective *p*-values of 0.032, 0.05 and 0.017) and *RPS7* showed a tendency to be affected by the sex (*p*-value = 0.057). These four genes were removed from the analysis for muscle tissue. 

### 3.3. Definition of the Most Stable Gene

**Figure 1:**
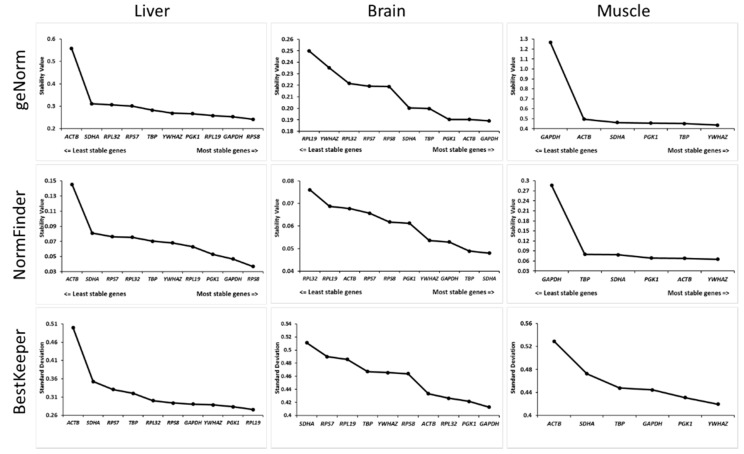
Stability values of the putative reference genes defined by three algorithms (geNorm, NormFinder and BestKeeper) for each tissue.

Gene stability was studied using three different algorithms: geNorm, NormFinder and BestKeeper. In liver, all genes tested were defined as stable because the stability values were below the recommendations (i.e., 1.5 for geNorm and 0.25 for NormFinder). Furthermore, the stability ranks defined by the three algorithms were very close and indicated *ACTB* as the least stable gene (Figure 1). *RPS8* and *RPL19* were the most stable genes according to geNorm and NormFinder. In brain, all genes passed the stability criteria (Figure 1). *GAPDH* was identified as the most stable gene by geNorm and BestKeeper, whereas *SDHA* was the most stable for NormFinder (Figure 1). In pectoral muscle, the analysis performed with NormFinder revealed that the *GAPDH* stability value was higher than the recommended value of 0.25, leading to its exclusion as a reference gene (Figure 1). The analysis performed with geNorm also indicated *GAPDH* as the least stable gene whereas the analysis based on BestKeeper revealed *ACTB* as the least stable gene. Nevertheless, all algorithms indicated *YWHAZ* as the most stable gene for this tissue (Figure 1). 

### 3.4. Identification of the Combination of the Most Stable Genes

Whereas the BestKeeper algorithm is used to define the best reference gene, NormFinder can define the combination of the two most stable reference genes in terms of geometric mean. In contrast, geNorm can be used to define the most stable combination of two or more reference genes. To gain insight into the similarities between NormFinder and geNorm, the most stable combinations of two reference genes defined by each algorithm were compared.

**Figure 2:**
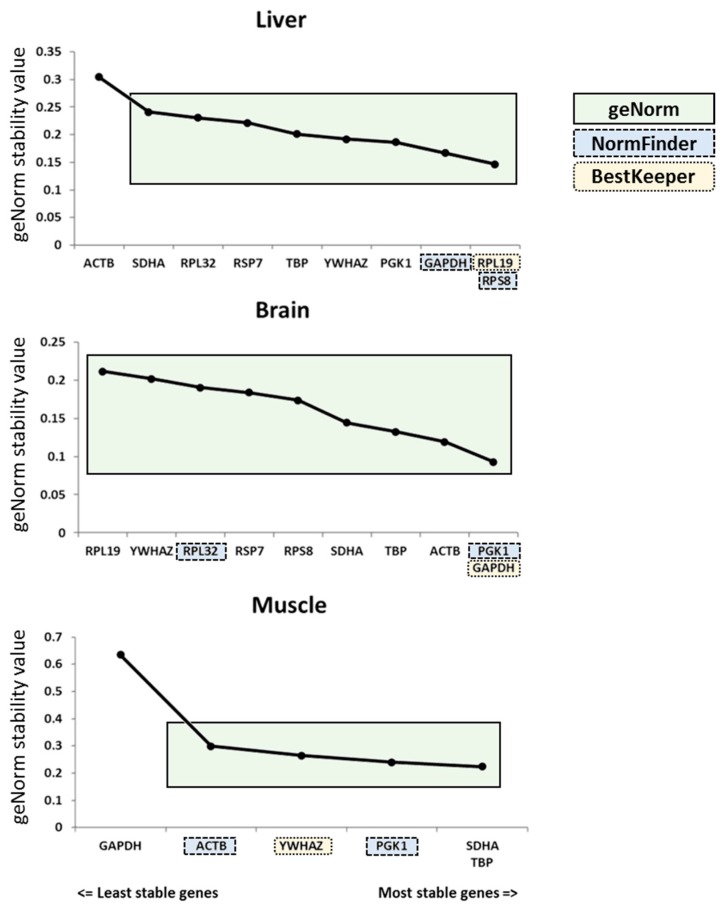
Reference gene combinations suitable for normalization defined by geNorm. The two most stable genes defined by geNorm are shown with one below the other at the right side of the graph. The green boxes indicate the combination of reference genes suitable for normalization as defined by geNorm. The blue boxes correspond to the genes defined as the most stable combination by NormFinder software. The yellow boxes correspond to the best reference genes selected by BestKeeper algorithm.

NormFinder revealed that the most stable pair of reference genes was *GAPDH* and *RPS8* for liver samples, *PGK1* and *RPL32* for brain samples and *PGK1* and *ACTB* for muscle samples (Table 4). Among all suitable genes, geNorm identified *RPL19* and *RPS8* as having the lowest stability values for liver samples, *PGK1* and *GAPDH* for brain samples and *SDHA* and *TBP* for muscle samples (Figure 2).

**Figure 3:**
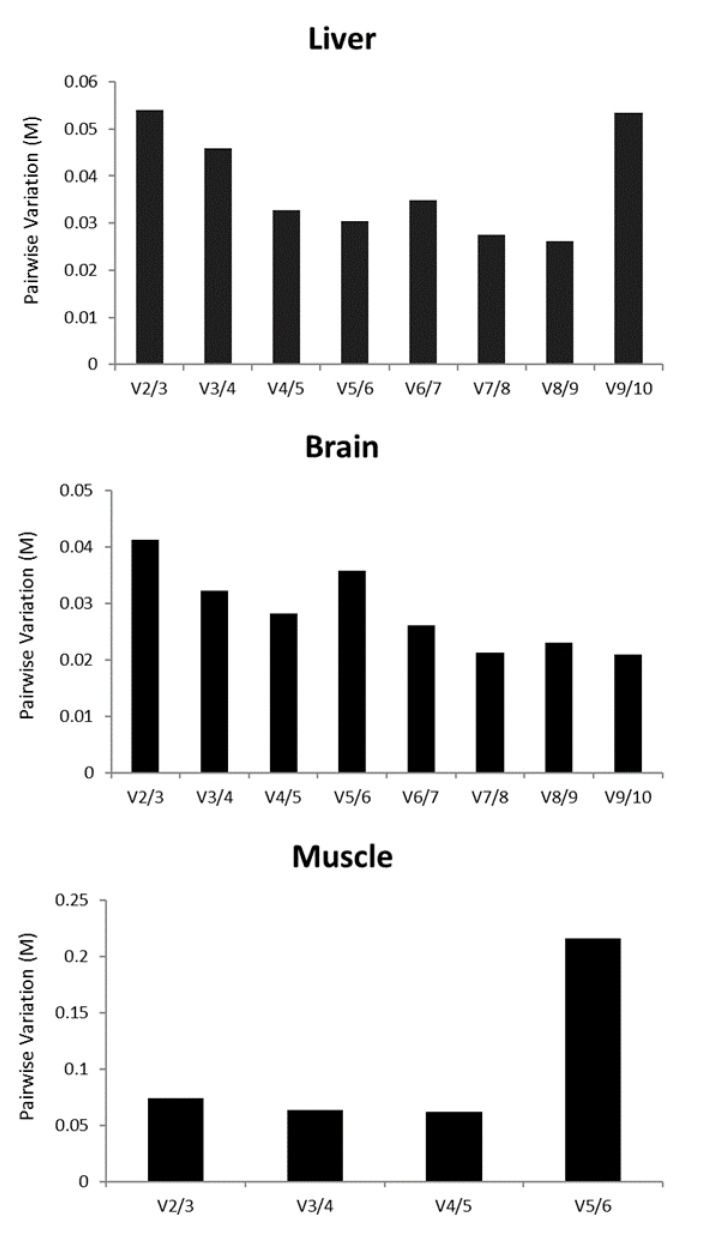
Analysis of the optimal number of reference genes for RT-qPCR normalization obtained by geNorm software. Pairwise variation (Vn/n+1) analysis was performed between the normalization factors (NF) NF n and NF n + 1. Each tissue was analyzed independently.

The geNorm algorithm allows the definition of the optimal combination of genes required for normalization. Therefore, we searched the optimal number of genes that was necessary for accurate normalization (Figure 2, green boxes, and Figure 3). The analysis revealed that the pairwise variation was the smallest with a combination of nine genes for the liver (all but *ACTB*) and all genes for the brain. For muscle tissue, five genes (*SDHA*, *TBP*, *PGK1*, *YWHAZ* and *ACTB*) were usable for normalization (Figure 3). 

### 3.5. Validation of Reference Genes

**Figure 4:**
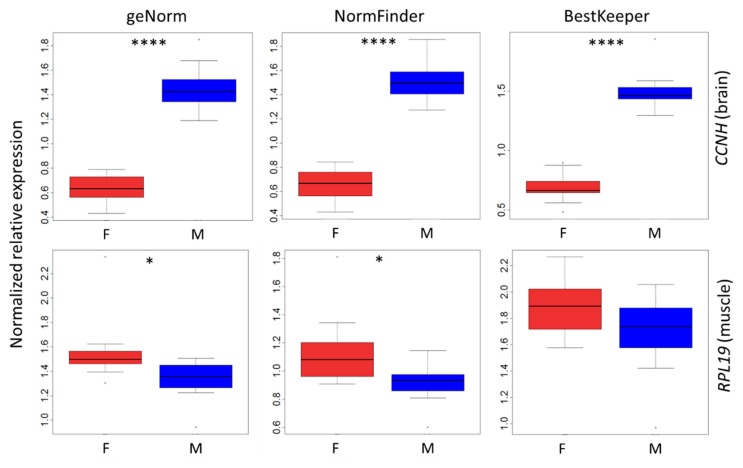
Expression of candidate genes normalized by each algorithm (geNorm, NormFinder and BestKeeper) in female (F) and male (M) quails. The expression of *CCNH* and *RPL19* was investigated in brain and in pectoral muscle, respectively. The reference genes defined by BestKeeper were *GAPDH* and *YWHAZ* in brain and in muscle, respectively. The combination of two reference genes identified by NormFinder was *PGK1* and *RPL32* in brain and *PGK1* and *ACTB* in muscle. The geNorm normalization factor was used, based on *GAPDH, ACTB, PGK1, TBP, SDHA, RPS8, RPS7, RPL32, YWHAZ* and *RPL19* in brain and *YWHAZ, TBP, PGK1, SDHA* and *ACTB* in muscle. * *p* value ≤ 0.05, **** *p*-value ≤ 0.0001.

To validate the reference gene groups defined by the three methods, we analyzed the expression of genes known to be differentially expressed between females and males (Figure 4). Two genes were selected, one for the brain based on the literature [2] (*CCNH*) and one for the muscle based on our previous analysis (*RPL19*; Table 3). Each gene was normalized with the reference gene group defined by each algorithm (Figure 2). We found that the expression of *CCNH* was significantly higher in female brains compared to male brains with all reference gene sets, with a fold-change >2 (Figure 4). In muscle, *RPL19* expression was significantly lower in males when using reference genes defined by geNorm and NormFinder with a fold-change of ~0.80 but not by that suggested by BestKeeper (Figure 4). 

## 4. Discussion

The analysis of gene expression is a common measurement in molecular studies and the current gold standard protocol is RT-qPCR [10,11,12,13,14]. However, the reliability of the results depends of the normalization. The choice of suitable reference genes is essential to allow the comparison of multiple samples [17]. Here, we defined and analyzed the stability of 10 putative reference genes in female and male Japanese quails in three different tissues (liver, brain and pectoral muscle). 

Based on the reference genes described in other animal model species [21,23,24,25,27,28,29,30,31,34], a set of 10 putative reference genes was defined (*ACTB, GAPDH, PGK1, RPS7, RPS8, RPL19, RPL32, SDHA, TBP, YWHAZ;* Table 1). The accuracy and efficiency of each primer couple were verified using serial RT dilutions (Table 2), melting curves (Supplementary Figures S1–S3) and Sanger sequencing for each tested tissue. All primers were suitable to analyze the transcript accumulation of their associated genes in all tissues (Table 2). 

The definition of reference genes has to take into account the effects studied such as sex (present study), aging, chemical treatment, disease or nutrition, among other factors [19,21,22]. Since we aimed to validate the reference genes by comparing the expression of candidate genes affected by the sex, we investigated the impact of sex on the raw expression of our putative reference genes. Whereas no effect of sex was observed for all genes tested in liver and brain, *RPL19*, *RPL32* and *RPS8* were significantly impacted in muscle and *RPS7* showed a tendency to be affected by the sex (*p* = 0.057; Table 3). Therefore, *RPL19*, *RPL32*, *RPS7* and *RPS8* genes may be considered only if the sex is unvarying. For instance, in another avian species, *RPL32* was reported as a suitable reference gene for breast muscle tissue of hens [29]. However, our analysis led to its exclusion as a reference gene. 

To assess reference gene stability, various algorithms are available. In this study, we used three different approaches based on the most cited algorithms in the literature [24,28]: geNorm [22], NormFinder [19] and BestKeeper [18]. In liver and brain, all candidate reference genes were identified as stable by all algorithms. However, in pectoral muscle, *GAPDH* was the least stable reference gene despite being described as a popular reference gene [12], leading to its exclusion as a reference gene in our study. Interestingly, our analysis showed that the most stable genes differed across tissues, confirming that a characterization of reference genes should be performed for each tissue of interest. Interestingly, no gene defined as the most stable was shared among all tissues, thus confirming the importance of the validation requirement for each experimental model [21]. Furthermore, the analysis of the stability ranks obtained with all three algorithms revealed that the rankings obtained with geNorm and NormFinder were closer in liver and pectoral muscle than that obtained with BestKeeper (Figure 1). This can be explained by the fact that both geNorm and NormFinder use ΔCq, in contrast to BestKeeper based on raw Cq [18]. This difference is in agreement with the previously reported similarity between geNorm and NormFinder in chicken [29]. Interestingly, this similarity is less clear for brain tissue, where the stability ranking of candidate reference genes differed between the three algorithms (Figure 1). This is likely due to the small variations of stability values obtained for each candidate reference gene. 

We validated the defined sets of genes by investigating their impact on the gene expression analysis of genes known to be differential between female and male quails. We analyzed *CCNH*, reported in the literature as sex-differential in adult quail brain samples [2], and *RPL19*, for which the analysis of the raw Cq revealed an impact of the sex in our experimental model in pectoral muscle (Table 3). The expression of *CCNH* was higher in male brains (more than 2-fold) compared to female brains regardless of the normalization method. Interestingly, the *RPL19* mRNA level was significantly lower in male muscles when geNorm and NormFinder methods were used (about ~0.8-fold), but not when BestKeeper normalization was performed. Given that the most gene stable gene was the same (*YWHAZ*) for all three algorithms in this tissue, this suggests that this difference is likely due to the number of genes used in the normalization. This is consistent with previous findings showing that at least two genes are advised when the differential studied is expected to be subtle [22]. Thus, our data supports the fact that multiple reference genes should be used to reveal low variations of candidate gene expression.

Based on stability values, our analysis showed that all 10 reference genes revealed by geNorm could be used to normalize gene expression in liver and brain in our biological context. For muscle tissue, five genes were shown to be suitable for normalization according to stability values. Our analysis also confirmed the similarity of results between NormFinder and geNorm, leading to the recommendation to use at least two reference genes to calculate the normalization factor. Thus, in our biological context, our data revealed that *GAPDH* and *RPS8* can be considered as good reference genes for liver samples, *PGK1* and *RPL32* for brain samples and *PGK1* and *ACTB* for pectoral muscle samples. The geNorm algorithm led to the identification of combinations of more reference genes in the three tissues that might of interest to reveal subtler gene expression changes, or that could be used in more complex biological setups. Nevertheless, we recommend performing a comprehensive reference gene analysis such as the one presented here each time a new experimental setup is used. 

## 5. Conclusions

Our study is the first attempt to identify reference genes in three tissues (liver, brain and muscle) of Japanese quail. As previously described in other species, our data revealed that the choice of reference genes highly depends on the experimental design as well as the algorithms used, and this requires fine-tuning. Nevertheless, our data describe suitable reference genes for brain, liver and pectoral muscle analyses in adult quails, leading to the recommendation to use NormFinder or geNorm as identification methods of reference genes and confirming the use of at least two reference genes to reveal subtle changes of candidate gene expression. This report could therefore be used as a guideline for the identification of reference gene sets in order to reinforce the reliability of RT-qPCR results.

## Figures and Tables

**Table 1:** Primers used in the study. F: forward primer. R: reverse primer.

Gene Symbol	Gene Name	Primer (5′–3′)	Accession Number	Amplicon Size (bp)
*ACTB*	*Actin β*	F: TGACCGCGGTACAAACACAG	XM_015876619.1	167
		R: CATACCAACCATCACACCCTGA		
*CCNH*	*Cyclin H*	F: GTCTGTAGTGGGAACGGCTT	XM_015849748.1	177
		R: TGTCCAACAGGGCTTTCTCG		
*GAPDH*	*Glyceraldehyde-3-phosphate dehydrogenase*	F: TCTCTGTTGTTGACCTGACCTG	XM_015873412.1	154
		R: ATGGCTGTCACCATTGAAGTC		
*PGK1*	*Phosphoglycerate kinase 1*	F: CAAGCTCACCCTGGACAAGT	XM_015860450.1	119
		R: GGACGGCTGCCTTGATTCTT		
*RPL19*	*Ribosomal protein L19*	F: GCATCGGTAAGAGGAAGGGT	XM_015885843.1	163
		R: ACGTTGCCCTTGACCTTCAG		
*RPL32*	*Ribosomal protein L32*	F: ATGGGAGCAACAAGAAGACA	XM_015875135.1	139
		R: TTGGAAGACACGTTGTGAGC		
*RPS7*	*Ribosomal protein S7*	F: TGTGGTGTTCATTGCTCAGAGA	XM_015859359.1	179
		R: TGCCATCCAGTTTTACGCGG		
*RPS8*	*Ribosomal protein S8*	F: GCTGACACCTGAGGAAGAAGA	XM_015870342.1	196
		R: CTTGCCTTCCAACACGTAGC		
*SDHA*	*Succinate dehydrogenase complex, subunit A*	F: TACGGGAAGGAAGGGGTTGT	XM_015854268.1	167
		R: CACAGTAGGCAGAACGGGAA		
*TBP*	*TATA box binding protein*	F: CCGGAATCATGGATCAGAAC	XM_015857924.1	85
		R: GGAATTCCAGGAGTCATTGC		
*YWHAZ*	*Tyrosine 3-monooxygenase/tryptophan 5-monooxygenase activation protein zeta*	F: CGAACAAAAGACGGAAGGCG	XM_015856086.1	154
		R: AACTTTGCTTTCTGCTTGCGA		

**Table 2:** Primer characteristics of the putative reference genes in liver, brain and muscle tissues. LDR: linear dynamic range (Cq min–Cq max). PCR eff.: PCR efficiency.

Tissue	Liver			Brain			Muscle		
Gene	LDR	PCR eff. (%)	R²	LDR	PCR eff. (%)	R²	LDR	PCR eff. (%)	R²
*ACTB*	18–27	93	0.98	17–28	98	0.99	19–28	98	0.96
*GAPDH*	17–26	99	0.99	16–26	103	0.99	14–22	95	0.98
*PGK1*	20–29	99	0.98	20–29	99	0.99	17–26	100	0.98
*RPL19*	19–28	101	0.95	20–30	106	0.99	20–29	97	0.96
*RPL32*	21–30	104	0.98	21–31	98	0.99	23–31	102	0.98
*RPS7*	20–29	103	0.97	20–29	105	0.99	22–30	99	0.97
*RPS8*	20–29	105	0.98	20–29	113	0.99	21–29	100	0.99
*SDHA*	22–31	98	0.98	20–29	106	0.99	21–30	101	0.98
*TBP*	19–34	102	0.97	20–29	93	0.99	25–34	99	0.94
*YWHAZ*	23–32	99	0.98	19–28	99	0.99	23–32	100	0.95

**Table 3:** *p*-values from *t*-test investigation of the impact of sex on reference gene expression.

Gene	Liver	Brain	Muscle
*ACTB*	0.099	0.213	0.751
*GAPDH*	0.363	0.254	0.800
*PGK1*	0.461	0.177	0.575
*RPL19*	0.780	0.726	0.032
*RPL32*	0.242	0.805	0.050
*RPS7*	0.775	0.635	0.057
*RPS8*	0.524	0.636	0.017
*SDHA*	0.829	0.401	0.258
*TBP*	0.916	0.322	0.155
*YWHAZ*	0.893	0.631	0.182

**Table 4:** Reference gene combination defined by NormFinder.

	Liver	Brain	Muscle
**Gene combination**	*GAPDH* and *RPS8*	*PGK1* and *RPL32*	*PGK1* and *ACTB*
**Stability value**	0.026	0.022	0.047

## Supplementary Materials

The following are available online at https://www.mdpi.com/2073-4425/10/3/197/s1, Figure S1: Specificity of primers tested by qRT-PCR in liver; Figure S2: Specificity of primers tested by qRT-PCR in pectoral muscle (*Pectoralis major*); Figure S3: Specificity of primers tested by qRT-PCR in brain.
