# Supplementary material for: Identification of Reference Genes for Quantitative Gene Expression Studies in Three Tissues of Japanese Quail
Source: Genes (Basel). 2019 Mar 4;10(3):197. doi: 10.3390/genes10030197 (PMC6470639; doi:10.3390/genes10030197)
Supplement: Supplementary file 1 [file genes-10-00197-s001.zip › VitorinoCarvalho_FigSupp1.pdf]

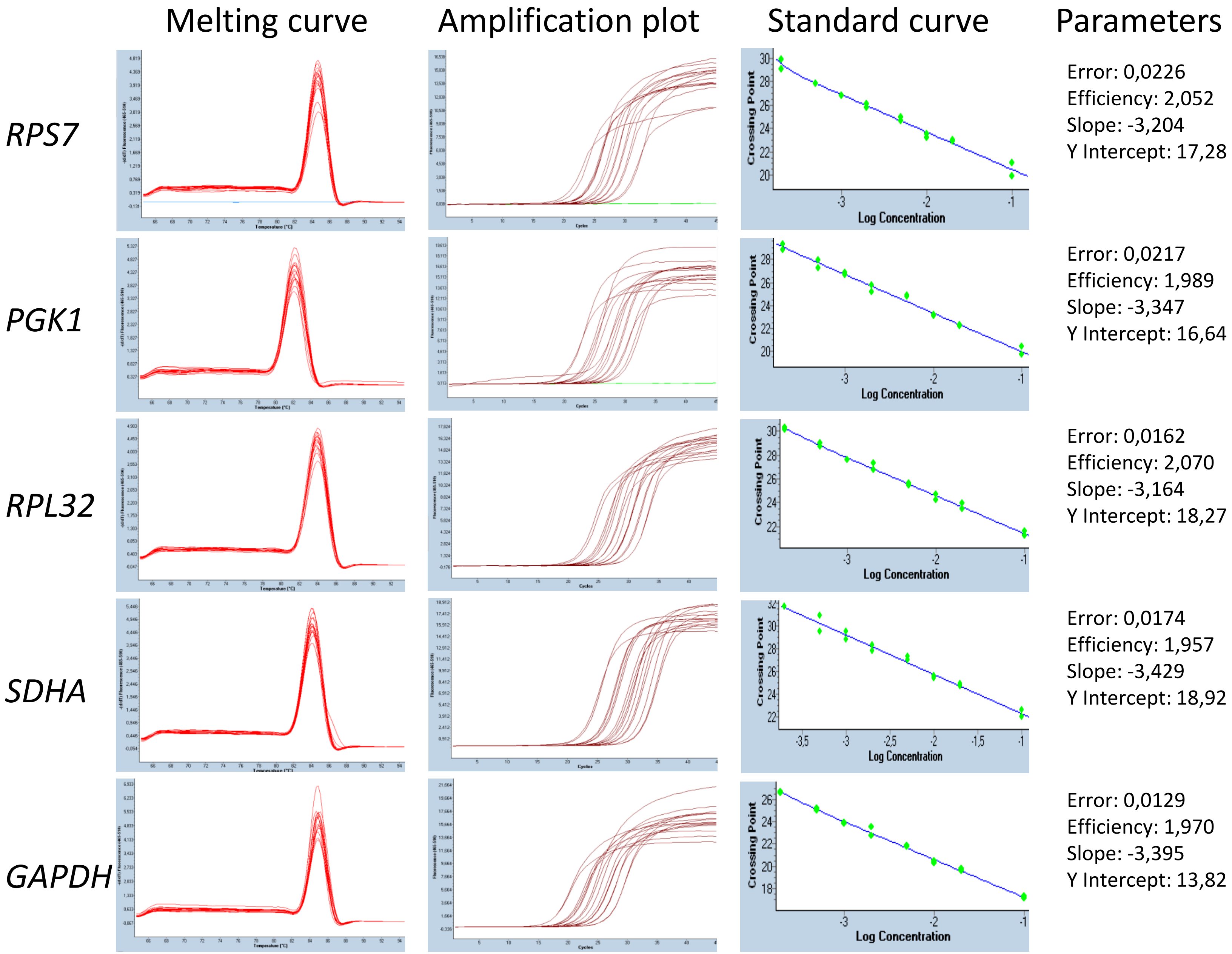
Melting curve
Amplification plot
Standard curve
Parameters
RPS7
Error: 0,0226
Efficiency: 2,052
Slope: -3,204
Y Intercept: 17,28
PGK1
Error: 0,0217
Efficiency: 1,989
Slope: -3,347
Y Intercept: 16,64
RPL32
Error: 0,0162
Efficiency: 2,070
Slope: -3,164
Y Intercept: 18,27
SDHA
Error: 0,0174
Efficiency: 1,957
Slope: -3,429
Y Intercept: 18,92
GAPDH
Error: 0,0129
Efficiency: 1,970
Slope: -3,395
Y Intercept: 13,82
Crossing Point
Log Concentration

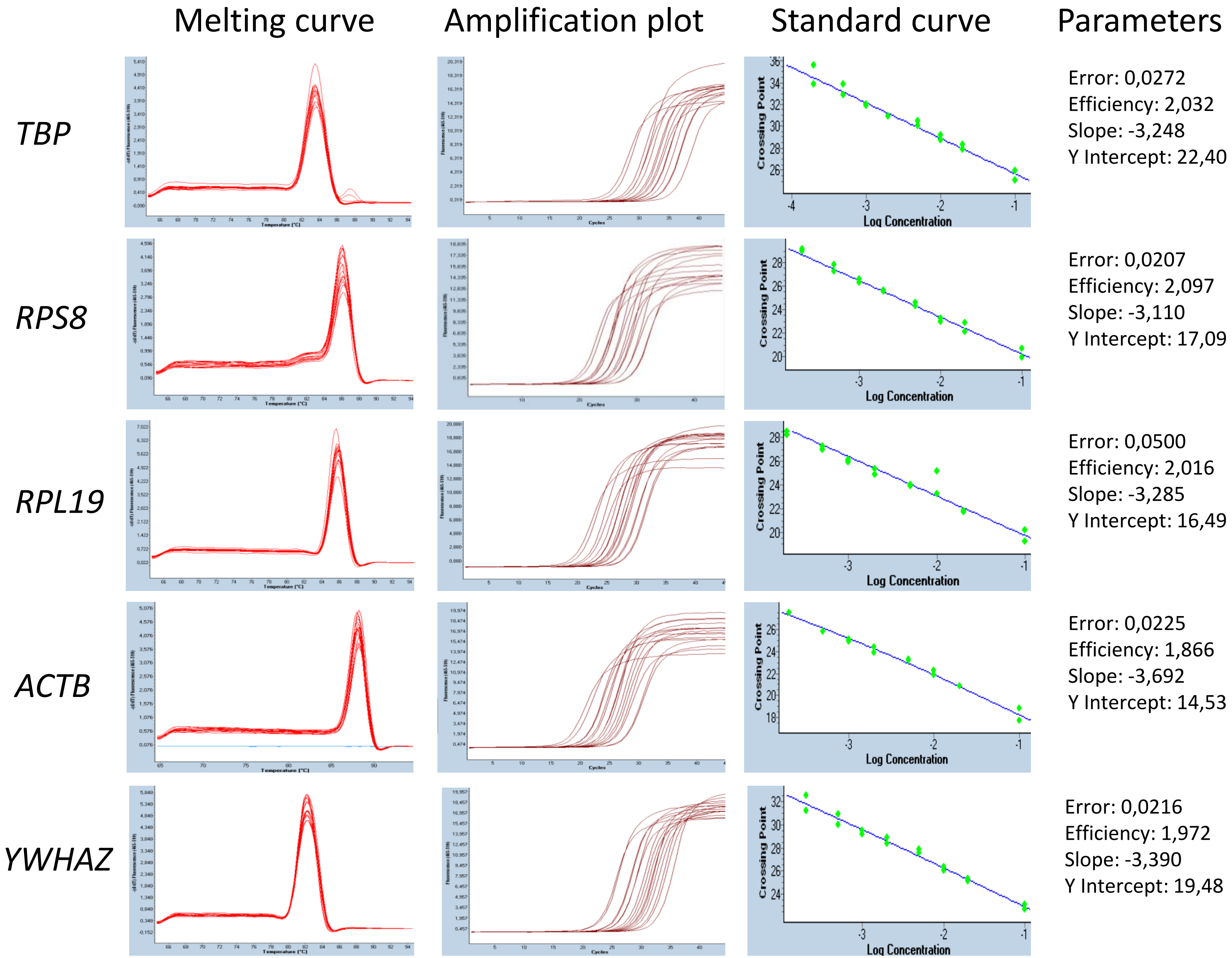
Melting curve
Amplification plot
Standard curve
Parameters
TBP
Error: 0,0272
Efficiency: 2,032
Slope: -3,248
Y Intercept: 22,40
RPS8
Error: 0,0207
Efficiency: 2,097
Slope: -3,110
Y Intercept: 17,09
RPL19
Error: 0,0500
Efficiency: 2,016
Slope: -3,285
Y Intercept: 16,49
ACTB
Error: 0,0225
Efficiency: 1,866
Slope: -3,692
Y Intercept: 14,53
YWHAZ
Error: 0,0216
Efficiency: 1,972
Slope: -3,390
Y Intercept: 19,48
Crossing Point
Log Concentration
