# Supplementary material for: Identification of Reference Genes for Quantitative Gene Expression Studies in Three Tissues of Japanese Quail
Source: Genes (Basel). 2019 Mar 4;10(3):197. doi: 10.3390/genes10030197 (PMC6470639; doi:10.3390/genes10030197)
Supplement: Supplementary file 1 [file genes-10-00197-s001.zip › VitorinoCarvalho_FigSupp2.pdf]

	Melting curve	Amplification plot	Standard curve	Parameters

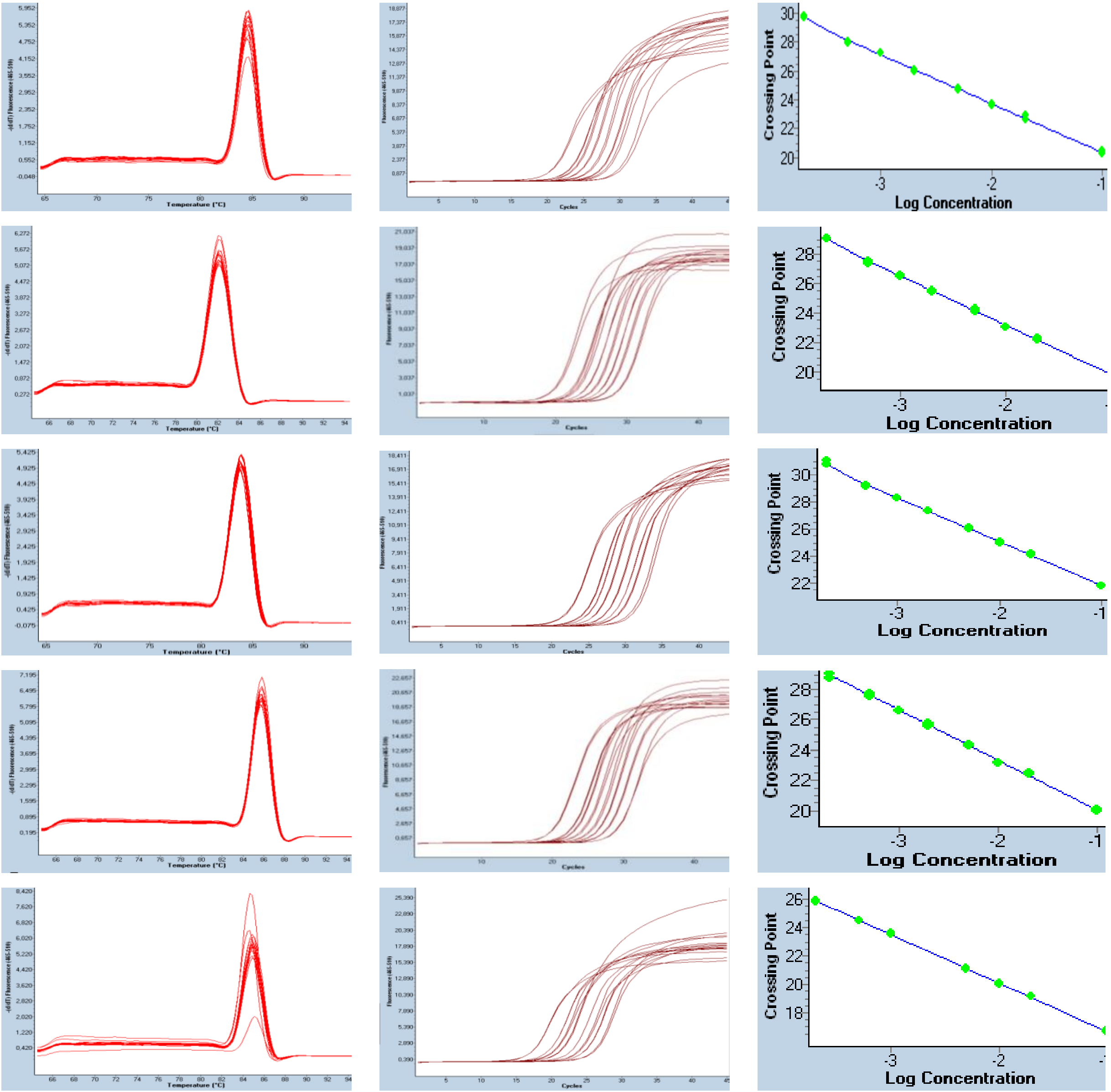

Gene	Parameters
RPS7	Error: 0,00953<br>Efficiency: 1,975<br>Slope: -3,384<br>Y Intercept: 16,96
PGK1	Error: 0,00491<br>Efficiency: 2,007<br>Slope: -3,306<br>Y Intercept: 16,55
RPL32	Error: 0,00569<br>Efficiency: 2,047<br>Slope: -3,213<br>Y Intercept: 18,61
SDHA	Error: 0,00943<br>Efficiency: 2,012<br>Slope: -3,294<br>Y Intercept: 16,70
GAPDH	Error: 0,00581<br>Efficiency: 1,980<br>Slope: -3,371<br>Y Intercept: 13,37

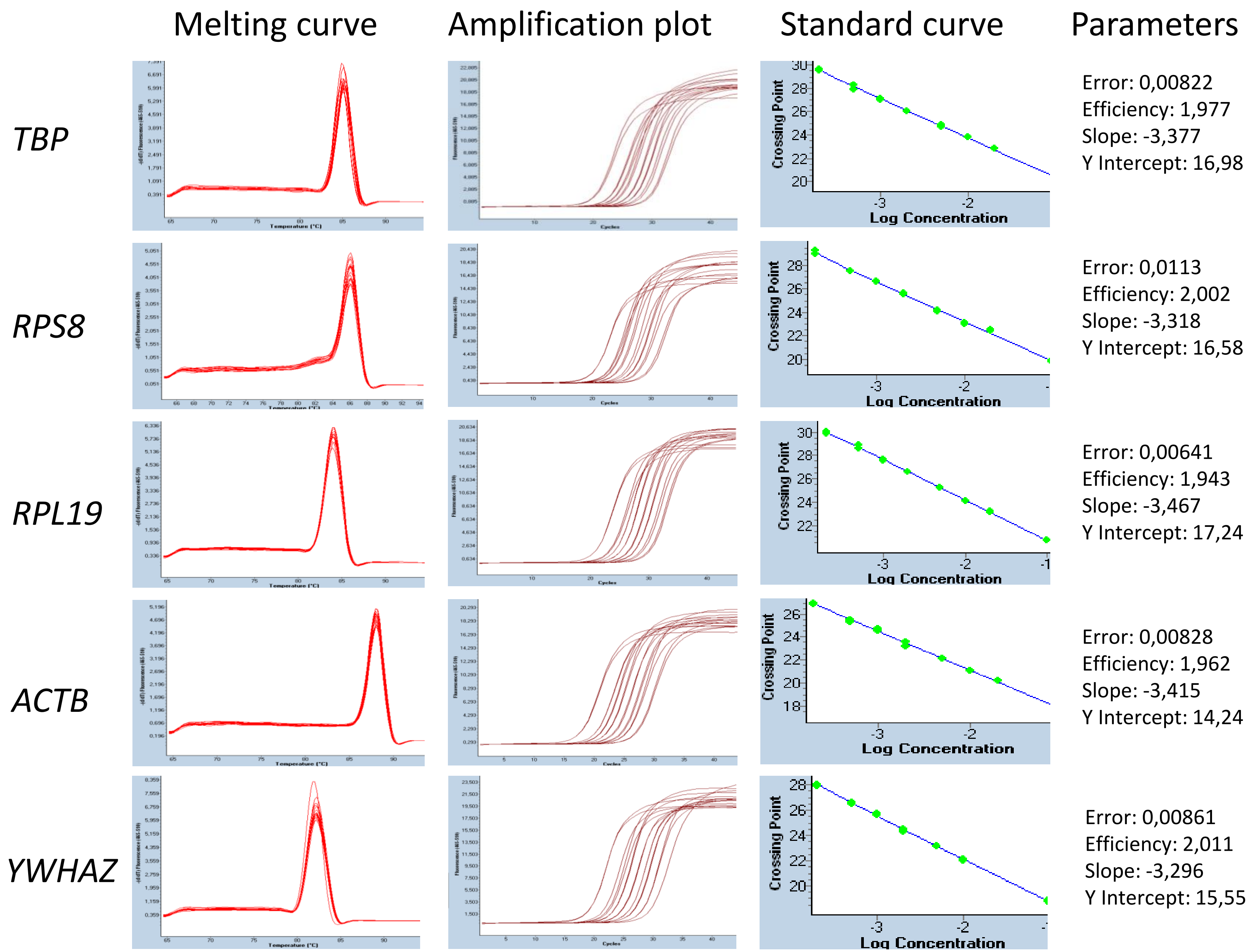
Melting curve
Amplification plot
Standard curve
Parameters
TBP
Error: 0,00822
Efficiency: 1,977
Slope: -3,377
Y Intercept: 16,98
RPS8
Error: 0,0113
Efficiency: 2,002
Slope: -3,318
Y Intercept: 16,58
RPL19
Error: 0,00641
Efficiency: 1,943
Slope: -3,467
Y Intercept: 17,24
ACTB
Error: 0,00828
Efficiency: 1,962
Slope: -3,415
Y Intercept: 14,24
YWHAZ
Error: 0,00861
Efficiency: 2,011
Slope: -3,296
Y Intercept: 15,55
Crossing Point
Log Concentration
Temperature (°C)
Cycles
