# Supplementary material for: Identification of Reference Genes for Quantitative Gene Expression Studies in Three Tissues of Japanese Quail
Source: Genes (Basel). 2019 Mar 4;10(3):197. doi: 10.3390/genes10030197 (PMC6470639; doi:10.3390/genes10030197)
Supplement: Supplementary file 1 [file genes-10-00197-s001.zip › VitorinoCarvalho_FigSupp3.pdf]

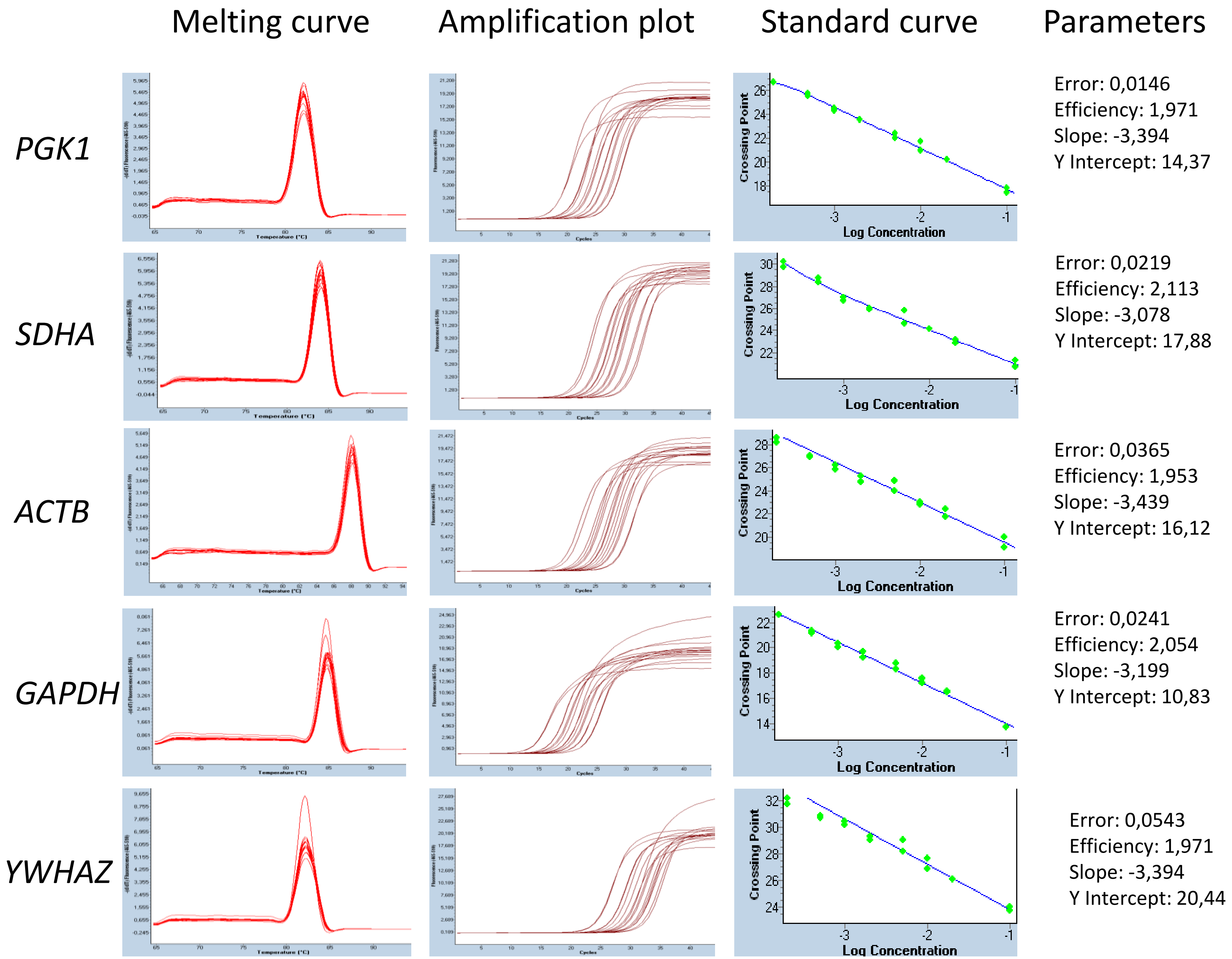
Melting curve
Amplification plot
Standard curve
Parameters
PGK1
Error: 0,0146
Efficiency: 1,971
Slope: -3,394
Y Intercept: 14,37
SDHA
Error: 0,0219
Efficiency: 2,113
Slope: -3,078
Y Intercept: 17,88
ACTB
Error: 0,0365
Efficiency: 1,953
Slope: -3,439
Y Intercept: 16,12
GAPDH
Error: 0,0241
Efficiency: 2,054
Slope: -3,199
Y Intercept: 10,83
YWHAZ
Error: 0,0543
Efficiency: 1,971
Slope: -3,394
Y Intercept: 20,44
Crossing Point
Log Concentration

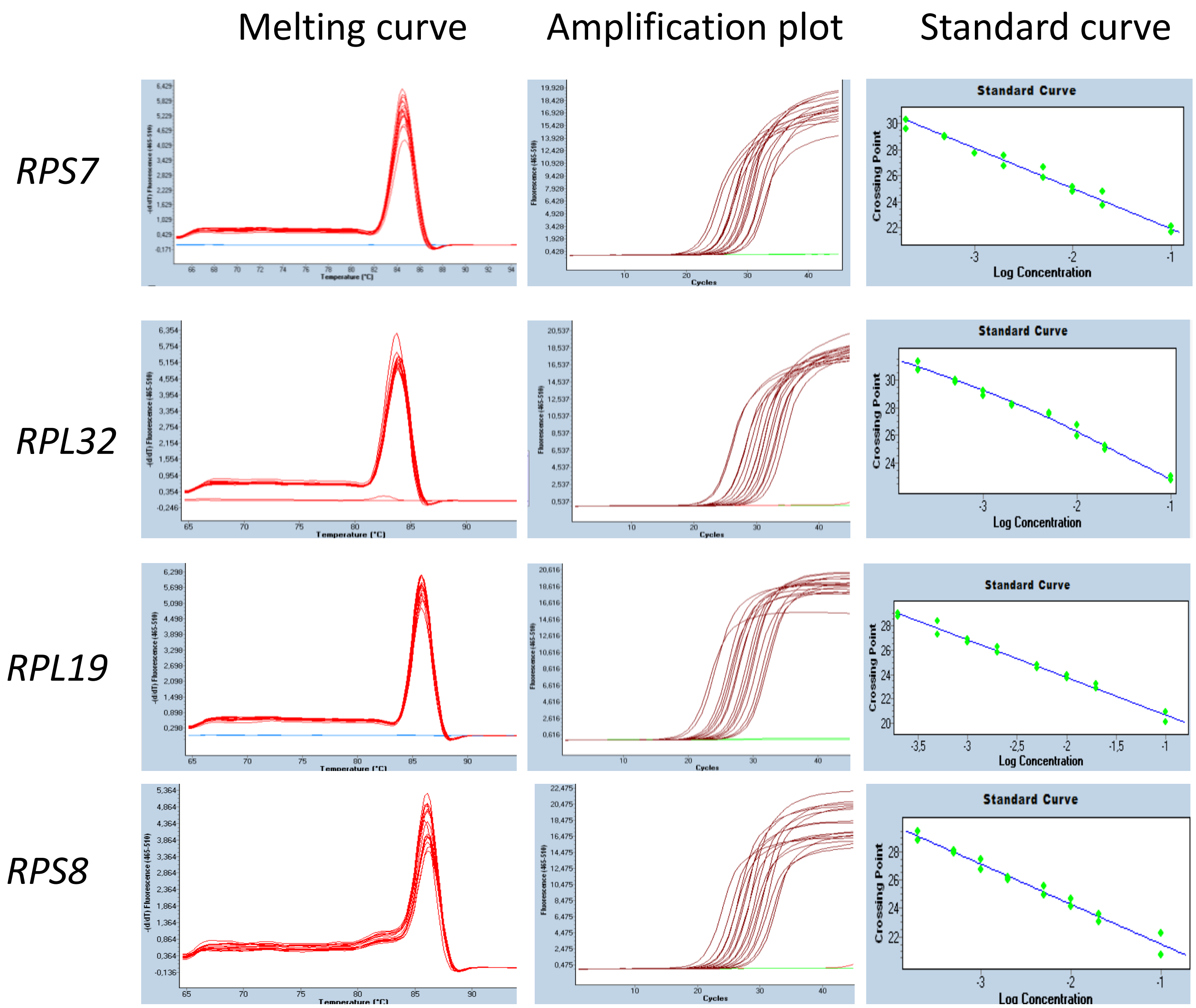

Parameters

RPS7
Error: 0,0246
Efficiency: 2,100
Slope: -3,103
Y Intercept: 18,82

RPL32
Error: 0,0205
Efficiency: 1,966
Slope: -3,406
Y Intercept: 19,45

RPL19
Error: 0,0413
Efficiency: 2,110
Slope: -3,083
Y Intercept: 17,60

RPS8
Error: 0,0117
Efficiency: 2,269
Slope: -2,811
Y Intercept: 18,68

Melting curve | Amplification plot | Standard curve | Parameters

TBP

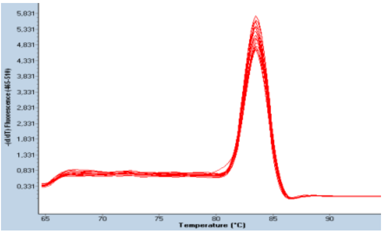

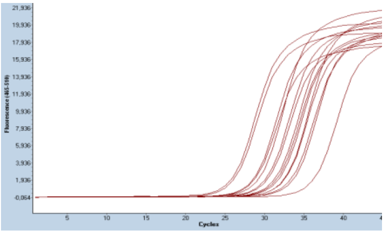

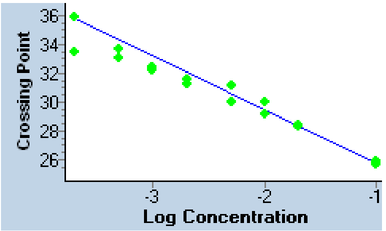

Error: 0,0611
Efficiency: 1,856
Slope: -3,723
Y Intercept: 22,00
